# Urgent Ultrasound Guided Hemodynamic Assessments by a Pediatric Medical Emergency Team: A Pilot Study

**DOI:** 10.1371/journal.pone.0066951

**Published:** 2013-06-25

**Authors:** David J. Zorko, Karen Choong, Jonathan Gilleland, Barbara Agar, Shawn Baker, Cindy Brennan, Eleanor Pullenayegum

**Affiliations:** 1 Faculty of Medicine, University of Ottawa, Ottawa, Ontario, Canada; 2 Department of Pediatrics, Faculty of Health Sciences, McMaster University, Hamilton, Ontario, Canada; 3 Department of Critical Care, Faculty of Health Sciences, McMaster University, Hamilton, Ontario, Canada; 4 Department of Clinical Epidemiology and Biostatistics, Faculty of Health Sciences, McMaster University, Hamilton, Ontario, Canada; National Institutes of Health, United States of America

## Abstract

**Purpose:**

To determine the feasibility of using the Ultrasound Cardiac Output Monitor (USCOM) as an adjunct during hemodynamic assessments by a pediatric medical emergency team (PMET).

**Methods:**

Pediatric in-patients at McMaster Children’s Hospital aged under 18 years requiring urgent PMET consultation, were eligible. Patients with known cardiac outflow valve defects, Pediatric Critical Care Unit in-patients, and those in cardiorespiratory arrest, were excluded. The primary outcome was feasibility, and the ease of USCOM transport and application as assessed by a self-administered user questionnaire. Secondary outcomes included the quality of USCOM measurements, and agreement in clinical versus USCOM-derived assessments.

**Results:**

Forty-one patients from 85 eligible PMET consultations were enrolled between March and August 2011. A total of 55 USCOM assessments were performed on 36 of 41 (87.8%) participants. USCOM could not be completed in 5 (12.2%) participants due to patient agitation (n = 4) and emergent care (n = 1). USCOM was reported as easy to transport and apply by 97.4% and 94.7% of respondents respectively, not obstructive to patient care by 94.7%, and yielded timely measurements by 84.2% respondents. USCOM tracings were of good quality in 41 (75.9%) assessments. Agreement between clinical and USCOM-derived hemodynamic assessments by two independent raters was poor (Rater 1: κ = 0.094; Rater 2: κ = 0.146).

**Conclusion:**

USCOM can be applied by a PMET during urgent hemodynamic assessments in children. While USCOM has been validated in stable children, its role in guiding hemodynamic resuscitation and informing therapeutic goals in a hemodynamically unstable pediatric population requires further investigation.

## Introduction

Timely recognition and early resuscitation of pediatric patients according to their hemodynamic physiology and goal-directed endpoints improves survival and functional outcomes in children with shock [1]–[4]. However, a clinician’s ability to estimate the hemodynamic status of children and infants based on the physical exam alone is poor [5]. There are currently several adjunctive methods of assessing a patient’s hemodynamic status, such as pulmonary artery catheters, pulse contour cardiac output, 2D echocardiography, and central venous oximetry [2], [6]. However, these tools are limited by their need for invasive access, inability to provide real-time measurements, the prerequisite expertise and setting required to conduct some of these measurements (i.e. an intensive care unit), and limited evidence of efficacy on clinically important outcomes [6]–[8]. Therefore, children requiring hemodynamic resuscitation outside the pediatric critical care unit (PCCU) remain primarily dependent on the clinical assessment. A reliable, non-invasive, objective method of assessing hemodynamic physiology that can be easily and rapidly applied in a broad range of children within and beyond the PCCU would be a valuable adjunct to optimizing the resuscitation of children in shock [9], [10].

The Ultrasound Cardiac Output Monitor (USCOM®; USCOM Ltd., Sydney, Australia) is a non-invasive bedside monitoring tool that utilizes continuous-wave Doppler ultrasound to measure hemodynamic parameters such as preload, systemic vascular resistance, cardiac index, and inotropy [11]. USCOM has been validated against echocardiography and cardiac catheter measurements in neonatal and pediatric normal cardiac anatomy populations, respectively [12]–[16]. This tool has been increasingly used to assess hemodynamic status in adult and pediatric critical care settings [9], [17]–[19], and continues to be a recommended method of monitoring therapeutic endpoints in the resuscitation of septic shock [9], [20]. However, its impact on patient important outcomes, and its use beyond the PCCU setting and by non-physician clinicians not been evaluated to date. The objective of this prospective observational pilot study was to evaluate the feasibility of using USCOM as an adjunctive tool during urgent hemodynamic assessments in children by members of a pediatric medical emergency team (PMET). We hypothesized that physician and non-physician PMET members could promptly apply USCOM during patient assessments and obtain USCOM measurements of similar quality. As there is currently no gold-standard method for the non-invasive measurement of hemodynamic endpoints in children, we also explored the agreement between USCOM-derived and clinician hemodynamic assessments.

## Materials and Methods

### Ethics Statement

The Faculty of Health Sciences/Hamilton Health Sciences Research Ethics Board approved a waived consent model for this study, given the minimal-risk and time-sensitive study design.

### Study Participants

This pilot study was conducted at McMaster Children’s Hospital (Hamilton, Canada) between March and August 2011. Consecutive in-patients at the institution aged 0 to 18 years, requiring urgent assessment by the PMET, were eligible. Patients with known valvular cardiac defects, admitted to the PCCU, or requiring “code blue” activation (i.e. cardiorespiratory arrest) or elective PMET follow-up assessments, were excluded. At the institution, a “Code Blue Team,” not the PMET, attends cardiorespiratory arrests.

### Intervention

The PMET at McMaster Children’s Hospital can be urgently consulted for critical patient events (Table S1) [21]. All PMET consultations are attended by the following: a registered nurse (RN), a registered respiratory therapist (RRT), a resident in training, and a critical care trained physician (PCCU fellow or attending). For the purposes of this study, 5 PMET members (2 attending physicians, 2 RRTs, and 1 RN) and the principle investigator (DZ) were trained in using USCOM [11]. The technique for obtaining USCOM measurements was adhered to as has been previously described [10].

In this study, USCOM measurements were performed at each eligible initial PMET patient consultation and in follow-up if clinically indicated. To minimize interference with patient management, USCOM was applied only after the clinical assessment by the PMET was completed. USCOM-derived measurements were available only to the USCOM operator, and were blinded to the study participants, healthcare staff, other PMET members, and the independent physician who provided a clinical impression of the patient’s hemodynamic status assessed just prior to the USCOM measurement. Demographic, clinical, and outcome data were recorded on standardized case report forms for each study participant.

### Outcomes

The primary outcome was feasibility, as assessed by: (a) ability to enrol the sample size over the anticipated study duration; (b) protocol adherence, as assessed by the proportion of eligible PMET consultations in which USCOM was successfully conducted; and (c) the ease of transporting and applying the USCOM urgently in participants, as assessed on a 7-point Likert scale self-administered questionnaire with optional commentary.

Secondary outcomes included a quality assessment of USCOM traces, and agreement between clinician and USCOM-derived assessments of the patient’s hemodynamic status. USCOM trace quality was assessed independently by two investigators (DZ, KC) using a previously published 12-point scoring tool [22]. The quality of USCOM traces was categorized as either good (≥8 points) or poor (<8 points). Disagreements were resolved by consensus. USCOM-derived hemodynamic status was assessed independently by two investigators (KC, JG) using age-dependant normal range values for cardiac index, stroke volume, and systemic vascular resistance (Table S2, Table S3) [23]–[25]. Using these parameters, hemodynamic status was categorized as either euvolemic, hypovolemic, cardiogenic, hyperdynamic, or indeterminate. Each USCOM-derived assessment was then compared by an independent investigator (DZ) to the blinded physician’s clinical impression of the patient’s hemodynamic status.

### Statistical Analyses

We estimated a convenience sample size of 40 patients over a period of 20 weeks, in order to demonstrate feasibility. This estimate was based on previous PMET data of approximately 37 new consultations per month, 57% of which occur during the day shift, and the availability of USCOM operators during these shifts. Descriptive statistics are presented as counts and percentages with 95% confidence interval (CI), means (standard deviation [SD]) or medians (minimum [min], maximum [max]) as appropriate. Agreement was assessed using the kappa statistic. Kappa values ≥0.40 represented moderate to good agreement. The quality of USCOM tracings between clinician groups was assessed using the Chi^2^ test, and an alpha = 0.05 (2-sided) level of statistical significance. All analyses were performed using SPSS version 20.0 (SPSS Inc., Chicago, IL). This study was reported in accordance with the Strengthening the Reporting of Observational Studies in Epidemiology (STROBE) statement [26].

## Results

**Figure 1:**
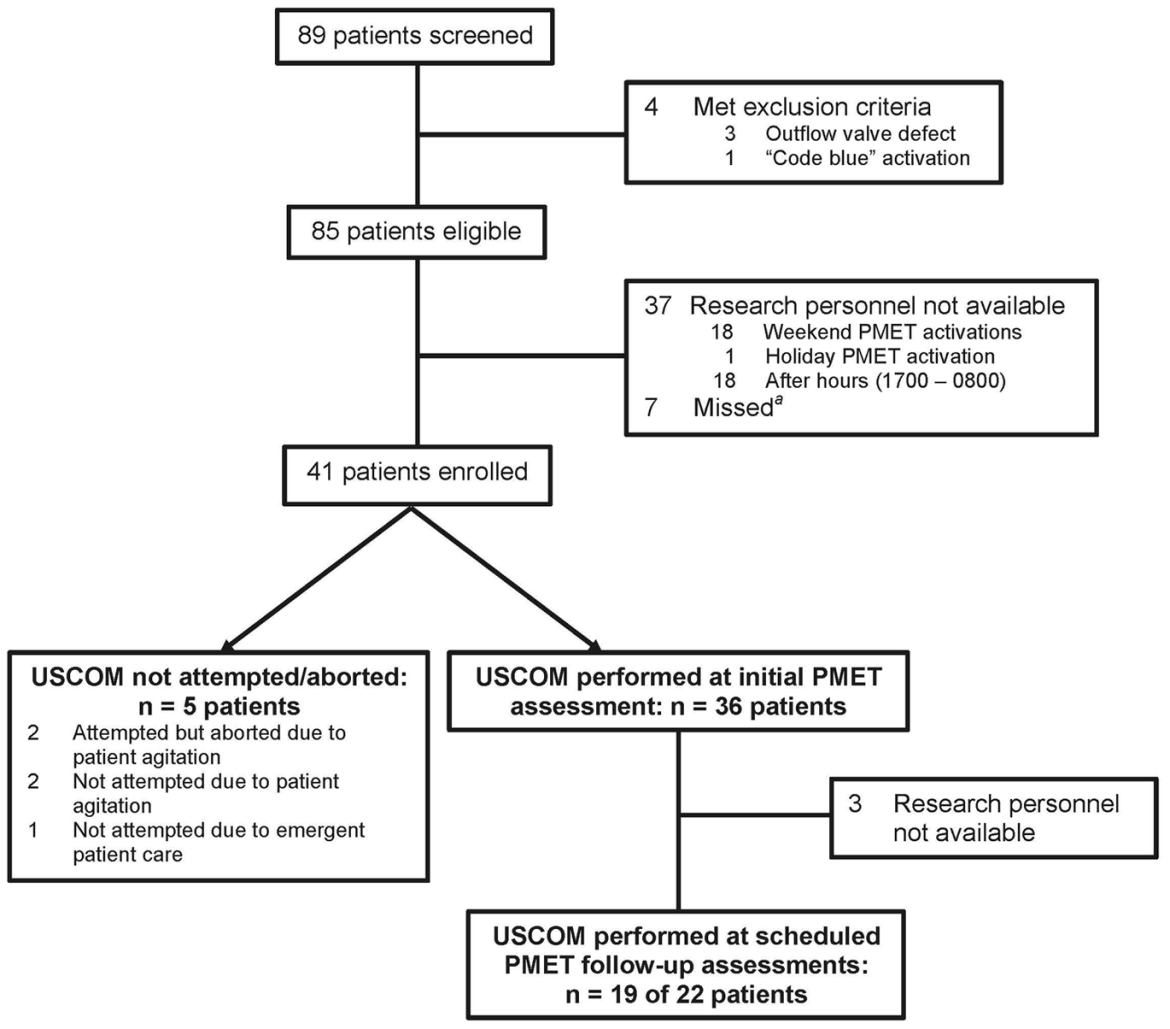
Enrolment and outcomes. USCOM indicates Ultrasound Cardiac Output Monitor; PMET, pediatric medical emergency team. *^a^*Missed patients indicates research personnel were available, but patient was not screened.

Eighty-nine PMET consultations were screened for eligibility during the 20-week study period between March and August 2011. Eighty-five patients met eligibility criteria, of which 41 (48.2%) were enrolled. Baseline characteristics of the study participants are presented in Table 1. Complete USCOM measurements were obtained during initial PMET assessments in 36 of the 41 (87.8%) participants, and in 19 of 22 (86.4%) scheduled follow-up visits (Figure 1). USCOM could not be conducted or was aborted in 5 (12.2%) participants because of patient agitation and emergency patient care. Of the 55 total USCOM assessments, 15 (27.3%) were conducted by a physician PMET member, 21 (38.2%) by RRT, 11 (20.0%) by the principal investigator, and 8 (14.5%) by RN.

**Table 1:** Baseline Characteristics.

Characteristic	Value^a^
Age in months	65.0 (70.6)
Weight in kg	19.6 (18.5)
Male sex, n (%)	18 (43.9)
Reason(s) for PMET consultation, n (%)	
Respiratory distress^b^	36 (87.8)
Hemodynamic instability^c^	22 (53.7)
Staff worried	19 (46.3)
Airway threat	7 (17.1)
Neurological deterioration^d^	4 (9.8)
Family worried	1 (2.4)
Other^e^	2 (4.9)
Admission Diagnosis, n (%)	
Respiratory tract infection	7 (17.1)
Sepsis	7 (17.1)
Bowel obstruction	6 (14.6)
Surgery	4 (9.8)
Encephalopathy	3 (7.3)
Chemotherapy	2 (4.9)
Inborn error of metabolism	2 (4.9)
Seizures	2 (4.9)
Other^f^	8 (19.5)
Vitals Signs at Initial PMET Consultation	
Temperature (°C)	37.7 (1.2)
Heart rate (bpm)	143 (37)
Systolic blood pressure (mmHg)	108 (21)
Respiratory rate (breaths/min)	40 (23)
Oxygen saturation (%)	97 (3)

PMET indicates pediatric medical emergency team.

a Data presented as mean (SD) unless otherwise specified.

b Indicates tachypnea, increased work of breathing, or hypoxemia.

c Indicates tachycardia, hypotension, or hypoperfusion.

d Indicates change in neurological status, decreased level of consciousness, or seizures.

e Other reasons for PMET consultation include lactic acidosis and hyperglycemia.

f Other admission diagnoses include multiple anomalies, apparent life-threatening event, trauma, sickle cell crisis, and dehydration.

The response rate for the user questionnaire was 92.7% (38/41). Three surveys were not completed early in the study when the USCOM was aborted as described above. Respondents’ impressions of applying USCOM as part of their assessments are presented in Table 2. The majority (97.4%; 37/38) of respondents found USCOM somewhat easy or easy to transport to the bedside, 94.7% (36/38) reported it was easy to apply, and 84.2% (32/38) felt that it yielded timely measurements. Only 5.3% (2/38) of respondents found USCOM somewhat obstructive to the patient care area. Respondents attributed unfavourable scores to the following reasons: patient agitation (n = 6), body habitus (kyphoscoliosis; n = 1), and waveform artefact in a patient on non-invasive mechanical ventilation (n = 1).

**Table 2:** Outcomes of Interest.

Characteristic	Value^a^	
Time of PMET arrival^b^ (hr:min)	0∶02 (0∶00,0∶10)	
Time taken to apply USCOM^c^ (hr:min)	0∶20 (0∶00,1∶56)	
User Questionnaire Scores^d^ (n = 38 respondents)		
Transportability to patient bedside	7 (4,7)	
Intrusiveness to patient care area	7 (1,7)	
Ease of operation	7 (1,7)	
Assessment timeliness	6 (1,7)	
USCOM Trace Quality Consensus Scores^e^, mean (SD)		
Overall	8.5 (1.4)	
By Assessment Category		
Initial assessments	8.5 (1.7)	
Follow-up assessments	8.5 (1.6)	
By Clinician Category		
MD	8.8 (1.7)	
RN	8.8 (1.6)	
RRT	8.3 (1.5)	
PI	8.1 (1.6)	
**Categorization of Hemodynamic Status based on USCOM (n = 54), n (%)**	**Rater 1**	**Rater 2**
Euvolemic circulation	23 (42.6)	12 (22.2)
Hypovolemic shock	10 (18.5)	21 (38.9)
Cardiogenic shock	0 (0.0)	2 (3.7)
Hyperdynamic shock	21 (38.9)	19 (35.2)
Indeterminate	0 (0.0)	0 (0.0)

USCOM indicates Ultrasound Cardiac Output Monitor; PMET, pediatric medical emergency team; MD, physician; RRT, registered respiratory therapist; PI, principal investigator; RN, registered nurse.

a Data presented as median (min,max) unless otherwise specified.

b Indicates elapsed time between PMET consultation and PMET arrival.

c Indicates elapsed time between PMET arrival and start of USCOM assessment.

d 7-Point Likert Scale.

e Maximum score of 12. Scores ≥8 points denotes a good quality tracing, <8 points denotes a poor quality tracing [22].

The quality scores for USCOM tracings are presented in Table 2. Fifty-four tracings were available for quality assessment, of which 41 (75.9%) were of good quality. Of the 13 (24.1%) that were rated as poor quality, 9 were attributed to patient-related factors (i.e. agitation, tachycardia), 1 due to waveform artefact as described above, and 3 were operator-related. There was no statistical difference in the quality of USCOM tracings according to clinician type (p = 0.850).

The categorization of hemodynamic status from USCOM-derived measurements for each of the two independent raters is presented in Table 2. The inter-rater agreement was moderate (κ = 0.442, 95% CI [0.273, 0.610]). However, agreement between hemodynamic status according to USCOM and clinical impression was poor (Rater 1: κ = 0.094, 95% CI [−0.110, 0.298]; Rater 2: κ = 0.146, 95% CI [−0.007, 0.299]).

## Discussion

Pilot studies are an important and underutilized prerequisite to larger scale interventional trials, particularly in the setting of urgent and critical care pediatrics [27]. Such studies can provide important information regarding the feasibility of executing intended trial procedures, protocol adherence, enrolment rates, appropriateness of proposed eligibility criteria and potential outcomes of interest, as well as safety data. Feasibility outcomes derived from pilot studies are crucial to inform the methods of adequately powered and rigorously designed interventional clinical trials [28]. Therefore, we conducted this pilot study to assess important feasibility outcomes prior to an anticipated future interventional study. The PMET environment is complex and assessments are time-sensitive. Hence it is important to evaluate the utility, portability, and applicability of a new monitoring device within this dynamic team environment to ensure that patient care and safety are maintained, prior to future efficacy studies.

This pilot study demonstrates the following key findings. First, USCOM can be applied during urgent hemodynamic assessments in pediatric patients outside of the PCCU environment, and the device can be easily transported and integrated in a PMET setting without interfering with patient care. Second, USCOM measurements can be acquired by trained physician and non-physician personnel. In this study, USCOM measurements obtained by physicians and non-physicians were of similarly good quality, and poor quality was most often attributed to patient-related factors. Third, there was only moderate agreement in the interpretation of hemodynamic status with USCOM-derived data, and poor correlation between the clinical impression of hemodynamic status and that derived from the USCOM.

This is the first study to our knowledge to evaluate the urgent use of USCOM for hospitalized children outside of a PCCU setting, and by non-physician clinicians. This has significant implications, given that early recognition and prompt reversal of cardiorespiratory decompensation improves clinical outcomes and survival amongst children with septic shock [29]–[31]. We hypothesized that both physician and non-physician PMET members can be trained in operating USCOM, and this study revealed that these PMET members demonstrated similar competency in acquiring USCOM measurements. This has potential importance if USCOM is ultimately shown to be a useful adjunct for monitoring therapeutic endpoints during resuscitation, as hemodynamic assessments can be conducted by physician or non-physician trained clinicians. Currently, some PMETs are not led by physician members [32], [33]. We also observed a higher proportion of good quality USCOM tracings and user satisfaction than previously reported [34].

We used previously published USCOM-derived hemodynamic variables for the classification of USCOM-derived hemodynamic status of the participants [23]–[25]. Both raters reported that assigning hemodynamic physiology based on USCOM in isolation of physical examination data was challenging. Along with a participant’s heart rate and blood pressure, additional significant physical findings were felt to be important to each rater’s overall impression, particularly if the patient was suspected to have compensated shock or other confounding factors that may influence heart rate, blood pressure, and perfusion. This likely contributed to the moderate agreement between raters in USCOM-derived classification of hemodynamic status. We used a limited number of key USCOM parameters to categorize hemodynamic status, which we felt were pragmatic [35], [36]. Including additional indices such as stroke volume responsiveness and Smith-Madigan Inotropy Index may have added supportive data to the assessments; however, the required software and pediatric reference ranges had not been validated at the time of our study.

An objective of this pilot study was to evaluate the correlation between clinical hemodynamic assessment and USCOM methods, rather than to validate which method is superior. The poor correlation between USCOM and clinical assessments may be explained by the following. First, there are limitations to USCOM. Pediatric USCOM validation studies were performed previously under more controlled conditions in healthy individuals, anesthetised or sedated patients in the operating room and PCCU [13], [14], [23]–[25]. As a result, it is unclear how reliable its measurements are in physiologically unstable or decompensating patients. Second, there are limitations to hemodynamic assessments based on clinical examination alone, which are well recognized [5], [9]. Third, both methods are limited when used in isolation, while the combination of information derived from both may increase the validity of hemodynamic assessments. Clinicians currently remain dependent on physical examination to provide “real-time” information on response to therapy. Adjunctive tools that can provide rapid, non-invasive information regarding a patient’s physiology may further optimize hemodynamic assessments and subsequent interventions. Only one published study to date suggests USCOM may be useful in decision-making regarding the choice of vasoactive drugs in children with septic shock [9]. However, as neither the clinical examination nor USCOM are established gold standards for hemodynamic measurement in children, the results of this pilot trial should be interpreted with caution. They rationalize the need for further validation and evidence to inform how such technology may be incorporated in goal-directed therapeutic targets within pediatric resuscitation guidelines [20].

The main limitation to enrolment in this pilot study was the availability of research personnel, which is not unique to this study. Nevertheless, we were able to apply the intervention to approximately half of the eligible patients and achieve our projected enrolment rate. Despite the reported ease of its use and transportability, there are potential barriers to the application of USCOM by the PMET. We observed that poor quality tracings and abandonment of USCOM assessment were most often related to patient distress or agitation, since noise (e.g. crying) and movement can alter USCOM measurements. Similar technical issues were observed in relation to patient body habitus, which can affect ideal patient positioning required to conduct an USCOM assessment. Waveform artefact from non-invasive mechanical ventilation has not been reported in the past and requires further study.

### Conclusions

Current evidence suggests that effective management of shock in pediatric in-patients outside of the critical care setting is hindered by limitations in the physical exam and available adjuncts to hemodynamic measurement. Non-invasive tools that can better rationalize and monitor resuscitative decision-making may therefore yield better outcomes for these children. The results of this study indicate that USCOM is a feasible adjunct for rapid hemodynamic assessment for pediatric patients; however, our preliminary observations regarding the discordance between the clinical assessment and USCOM-derived measurements strongly support the need for further research. While USCOM is attractive as a rapid non-invasive tool to guide our clinical decision-making, it requires further evaluation of its utility and validity as an adjunct to monitoring goal-directed therapy, and its correlation with patient important outcomes.

## Supporting Information

Table S1 McMaster Children’s Hospital Pediatric Medical Emergency Team activation guidelines. SaO_2_ indicates oxygen saturation; FiO_2_, fraction of inspired oxygen.
(DOC)

Table S2 Categorization of Hemodynamic Status. Hemodynamic status was assessed in the context of abnormal vital signs, with the understanding that MAP may be normal in compensated shock, and heart rate and blood pressure may be elevated due to non-circulatory causes [35], [36]. MAP indicates mean arterial pressure; HR, heart rate; CI, cardiac index; SVRI, systemic vascular resistance index; SV, stroke volume.
(DOC)

Table S3 USCOM-derived hemodynamic reference ranges in healthy children [23]–[25]. HR indicates heart rate; SV, stroke volume; CI, cardiac index; SBP, systolic blood pressure; SVRI, systemic vascular resistance index.
(DOC)
