# Supplementary material for: Urgent Ultrasound Guided Hemodynamic Assessments by a Pediatric Medical Emergency Team: A Pilot Study
Source: PLoS One. 2013 Jun 25;8(6):e66951. doi: 10.1371/journal.pone.0066951 (PMC3692535; doi:10.1371/journal.pone.0066951)
Supplement: Table S1 — McMaster Children’s Hospital Pediatric Medical Emergency Team activation guidelines. SaO2 indicates oxygen saturation; FiO2, fraction of inspired oxygen. (DOC) [file pone.0066951.s001.doc]

**Table S1.**

| **Clinical Suspicion** | | | |
| --- | --- | --- | --- |
| Nurse, physician or family member worried about clinical state | | | |
| **Airway** | | | |
| Threatened or obstructive symptoms: stridor, excessive secretions | | | |
| **Breathing** | | | |
| Severe respiratory distress, apnea, tachypnea or cyanosis | | | |
| Age | Respiratory Rate/min | Hypoxemia | |
| Term-3 months | >60 | SaO_2_ < 90% in >40% FiO_2_ | |
| 4-12 months | >50 |  | |
| 1-4 years | >40 | SaO_2_ < 60% in > 40% FiO_2_ | |
| 5-12 years | >30 | (cyanotic heart disease) | |
| 12 years + | >30 |  | |
| **Circulation** | | | |
| Age | Bradycardia | Tachycardia | Blood Pressure |
|  | (beats/min) | (beats/min) | (systolic mmHg) |
| Term-3 months | <100 | >180 | <50 |
| 4-12 months | <100 | >180 | <60 |
| 1-4 years | <90 | >160 | <70 |
| 5-12 years | <80 | >140 | <80 |
| 12 years + | <60 | >130 | <90 |
| **Neurologic State** | | | |
| Acute change in neurologic status or convulsion | | | |
