# Supplementary material for: Urgent Ultrasound Guided Hemodynamic Assessments by a Pediatric Medical Emergency Team: A Pilot Study
Source: PLoS One. 2013 Jun 25;8(6):e66951. doi: 10.1371/journal.pone.0066951 (PMC3692535; doi:10.1371/journal.pone.0066951)
Supplement: Table S2 — Categorization of Hemodynamic Status. Hemodynamic status was assessed in the context of abnormal vital signs, with the understanding that MAP may be normal in compensated shock, and heart rate and blood pressure may be elevated due to non-circulatory causes [35], [36]. MAP indicates mean arterial pressure; HR, heart rate; CI, cardiac index; SVRI, systemic vascular resistance index; SV, stroke volume. (DOC) [file pone.0066951.s002.doc]

**Table S2.**

| **Hemodynamic Status** | **MAP** | **HR** | **CI** | **SVRI** | **SV** |
| --- | --- | --- | --- | --- | --- |
| **Euvolemic circulation** | Normal | Normal | Normal | Normal | Normal |
| **Cardiogenic shock** | Low | High | Low | High | Low |
| **Hypovolemic shock** | Low | High | Low | High | Low |
| **Hyperdynamic shock** | Low | High | High | Low | Initially low/variable |
