# Supplementary material for: Urgent Ultrasound Guided Hemodynamic Assessments by a Pediatric Medical Emergency Team: A Pilot Study
Source: PLoS One. 2013 Jun 25;8(6):e66951. doi: 10.1371/journal.pone.0066951 (PMC3692535; doi:10.1371/journal.pone.0066951)
Supplement: Table S3 — USCOM-derived hemodynamic reference ranges in healthy children [23]–[25]. HR indicates heart rate; SV, stroke volume; CI, cardiac index; SBP, systolic blood pressure; SVRI, systemic vascular resistance index. (DOC) [file pone.0066951.s003.doc]

**Table S3.**

| **Age** | | **HR** | **SV** | **CI** | **SBP** | **SVRI** |
| --- | --- | --- | --- | --- | --- | --- |
| **0** | *Mean* |  | 14.8 | 4.38 | 85 | 1191 |
|  | *Min, Max* | 110, 140 | 12.91, 16.65 | 3.69, 5.07 | 68, 102 | 919, 1464 |
| **1** | *Mean* |  | 19.8 | 4.64 | 90 | 1125 |
|  | *Min, Max* | 110, 140 | 13.20, 23.06 | 3.55, 5.18 | 73, 107 | 904, 1345 |
| **2** | *Mean* |  | 29.1 | 4.98 | 96 | 1120 |
|  | *Min, Max* | 85, 115 | 23.01, 35.20 | 4.14, 5.81 | 76, 116 | 884, 1356 |
| **3** | *Mean* |  | 35.3 | 5.08 | 102 | 1166 |
|  | *Min, Max* | 85, 115 | 28.36, 42.15 | 4.08, 6.07 | 80, 124 | 876, 1457 |
| **4** | *Mean* |  | 40.4 | 5.18 | 102 | 1107 |
|  | *Min, Max* | 85, 115 | 33.54, 47.31 | 4.12, 6.24 | 81, 122 | 890, 1323 |
| **5** | *Mean* |  | 44.7 | 4.90 | 103 | 1176 |
|  | *Min, Max* | 75, 105 | 37.39, 51.99 | 4.09, 5.71 | 79, 126 | 947, 1405 |
| **6** | *Mean* |  | 49.3 | 4.77 | 107 | 1269 |
|  | *Min, Max* | 75, 105 | 40.64, 57.96 | 3.90, 5.63 | 82, 132 | 1014, 1525 |
| **7** | *Mean* |  | 53.8 | 4.81 | 111 | 1290 |
|  | *Min, Max* | 75, 105 | 43.63, 63.90 | 3.96, 5.67 | 87, 135 | 1073, 1507 |
| **8** | *Mean* |  | 59.1 | 4.81 | 114 | 1343 |
|  | *Min, Max* | 75, 105 | 47.97, 70.20 | 3.85, 5.78 | 90, 137 | 1078, 1607 |
| **9** | *Mean* |  | 62.3 | 4.69 | 113 | 1373 |
|  | *Min, Max* | 75, 105 | 51.22, 73.48 | 3.77, 5.62 | 90, 136 | 1121, 1625 |
| **10** | *Mean* |  | 70.0 | 4.46 | 115 | 1491 |
|  | *Min, Max* | 65, 100 | 56.17, 83.92 | 3.50, 5.43 | 92, 139 | 1116, 1867 |
| **11** | *Mean* |  | 73.8 | 4.45 | 117 | 1498 |
|  | *Min, Max* | 65, 100 | 60.60, 87.09 | 3.59, 5.31 | 94, 140 | 1181, 1815 |
| **12** | *Mean* |  | 86.0 | 5.13 | 122 | 1323 |
|  | *Min, Max* | 65, 100 | 71.32, 100.62 | 4.28, 5.98 | 106, 139 | 1090, 1556 |
| **13 - 16** | *Mean* |  | 92.3 | 4.61 | 124 | 1476 |
|  | *Min, Max* | 65, 100 | 79.40, 105.20 | 3.67, 5.55 | 103, 145 | 1102, 1850 |
